# Modelling the spread of *Wolbachia* in spatially heterogeneous environments

**DOI:** 10.1098/rsif.2012.0253

**Published:** 2012-06-06

**Authors:** Penelope A. Hancock, H. Charles J. Godfray

**Affiliations:** 1 Department of Zoology, University of Oxford, South Parks Road, Oxford, OX1 3PS, UK; 2 School of Life Sciences, University of Warwick, Gibbet Hill Road, Coventry, CV4 7AL, UK

**Keywords:** metapopulation model, symbiotic bacteria, reaction–diffusion model, mosquito-borne disease, density-dependence, travelling wave

## Abstract

The endosymbiont *Wolbachia* infects a large number of insect species and is capable of rapid spread when introduced into a novel host population. The bacteria spread by manipulating their hosts' reproduction, and their dynamics are influenced by the demographic structure of the host population and patterns of contact between individuals. Reaction–diffusion models of the spatial spread of *Wolbachia* provide a simple analytical description of their spatial dynamics but do not account for significant details of host population dynamics. We develop a metapopulation model describing the spatial dynamics of *Wolbachia* in an age-structured host insect population regulated by juvenile density-dependent competition. The model produces similar dynamics to the reaction–diffusion model in the limiting case where the host's habitat quality is spatially homogeneous and *Wolbachia* has a small effect on host fitness. When habitat quality varies spatially, *Wolbachia* spread is usually much slower, and the conditions necessary for local invasion are strongly affected by immigration of insects from surrounding regions. Spread is most difficult when variation in habitat quality is spatially correlated. The results show that spatial variation in the density-dependent competition experienced by juvenile host insects can strongly affect the spread of *Wolbachia* infections, which is important to the use of *Wolbachia* to control insect vectors of human disease and other pests.

## 1. Introduction

It has been estimated that up to 60 per cent of all insect species may be infected with the endosymbiotic bacteria *Wolbachia* [1]*. Wolbachia* are vertically transmitted from mothers to offspring, and can spread rapidly when introduced into a novel host population. The bacteria increase in frequency by manipulating their hosts' reproductive system, often using a mechanism known as cytoplasmic incompatibility (CI) [2,3]. CI causes incompatibility between the sperm of *Wolbachia-*infected males and the eggs of uninfected females, leading to failure of embryonic development unless the egg also carries the bacterium [2,4]. The presence of *Wolbachia* carrying males reduces the proportion of viable offspring that are uninfected, allowing the bacterium to spread. Therefore, *Wolbachia* epidemiology is closely linked to the host's demography and patterns of contact between individual hosts.

Currently, *Wolbachia* is being actively investigated as a potential tool to help control certain mosquito-borne diseases. In the mosquito *Aedes aegypti*, which is a vector of important human pathogens, including the dengue, yellow fever and the chikungunya viruses, *Wolbachia* infection can inhibit the development and transmission of human pathogens [5–8]. The bacteria can also reduce mosquito lifespan, which can have a strong impact on the pathogen transmission rate [9]. Proof of principle field releases of *Wolbachia* into wild populations of *A. aegypti* has recently been conducted [10,11]. However, mosquitoes have a complex life cycle in which the adult stage is preceded by juvenile stages that live in completely different microhabitats. Mosquito population dynamics depend on the availability of larval breeding habitat, which can be highly variable in space and time [12–18]. Density-dependent population regulation is also thought most likely to occur in the larval stage [19]. Models that represent these demographic processes are needed to understand better the dynamics of *Wolbachia* infection in these mosquito populations.

Reaction–diffusion models have been used to analyse the spatial dynamics of *Wolbachia* infection [20–22]. This approach assumes that the fitness advantage of *Wolbachia-*carriers in a particular location depends on the local infection frequency, the strength of CI as measured by offspring mortality in incompatible matings, and the fitness costs incurred through *Wolbachia* infection. If the spatial movement of the host population is assumed to be governed by a diffusion process in one dimension (see [21] and the electronic supplementary material) and if the abundance of hosts is assumed constant in space and time, the reaction–diffusion model can be solved to give a simple formula for the equilibrium speed (*v,* measured per host generation) of a travelling wave of *Wolbachia* infection,1.1
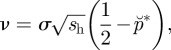
where *σ* is the standard deviation of the distance between the birthplace of parents and their offspring and *s*_h_ is the fraction of offspring resulting from an incompatible mating that fails to develop [21,23]. It is assumed that *Wolbachia* infection reduces host fecundity, by a proportion *s*_f_, and 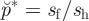. The quantity 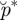 represents the trade-off between the fitness costs of *Wolbachia* infection and the fitness advantage afforded by CI. Equation (1.1) shows that if 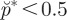 a *Wolbachia* infection that has become established in one location will spread spatially.

While reaction–diffusion models allow the derivation of the equilibrium travelling wave solution, they do not take into account important aspects of the demography of the host population that may affect spread [21,23–25]. In particular, the assumption of constant host population abundance restricts their ability to explore how host population dynamic processes interact with the dynamics of *Wolbachia* infection. For example, the introduction of *Wolbachia* can strongly affect the size of the local host population due to the action of CI, which can have implications for the spread of the infection. In many insect host populations, including mosquito species that are a target for *Wolbachia*-based vector control interventions, host age structure and density-dependent population regulation are important determinants of their dynamics. Non-spatial models of *Wolbachia* dynamics have demonstrated that these processes can strongly influence *Wolbachia* spread. For example, the introduction of insects infected with *Wolbachia* into a novel host population perturbs juvenile host density and the level of juvenile density-dependent competition, which affects the rate of insect introduction that is necessary to trigger spread [26,27].

In this study, a metapopulation model is developed to explore how the demography and dynamics of the host population affects *Wolbachia* spread. A key difference between this modelling approach and the reaction–diffusion model is that the host population size is dynamic and regulated in each subpopulation of the metapopulation (which we shall refer to as a patch) by juvenile density-dependent competition*.* The model also accounts for host age-structure, age-specific effects of *Wolbachia* infection on the host's demography and imperfect maternal transmission of *Wolbachia*. We first assess how well the analytic solution of the reaction–diffusion model equilibrium predicts properties of the spatial dynamics of the metapopulation model, including the equilibrium speed of the travelling wave of *Wolbachia* infection and the conditions for spatial spread to occur. We then explore the influence of spatial heterogeneity in larval carrying capacity on the spread of *Wolbachia*. To produce results that are most relevant to the application of *Wolbachia* to controlling mosquito-borne diseases, the model is parametrized to represent the demography of a mosquito population. Realistic features of the mosquito habitat are incorporated, including spatially correlated stochastic variation in larval habitat quality.

## 2. Model development

An age-structured metapopulation model was developed to explore the spatial dynamics of *Wolbachia* infection in an insect host population. The host population is subdivided into an array of patches connected by migration. A demographic model describes the host population dynamics in each patch. Values of the model parameters are as given in table 1, unless otherwise specified.

**Table 1.** The parameters used in the model and their default values.

symbol	definition	value
*σ*	standard deviation of the distance in number of patches between the birthplace of parents and offspring	2.7
*N*	number of patches	150
*λ*	daily female fecundity for insects uninfected with *Wolbachia*	30
*T*_L_	development time in days of larvae	10
*μ*, *α*, *β*	parameters of the density-dependent larval mortality function for larvae (see text and [26])	0.1,0.1, 0.2
*ω*	fraction of uninfected larvae produced by an infected adult female	0
*s*_h_	fraction of offspring of an uninfected female that fail to develop from an incompatible mating	0.99
*s*_f_	proportional reduction in fecundity due to *Wolbachia* carriage	0.05
*c, *γ*, r*	parameters of the mortality function for uninfected adults [26]	0.1, 1.0, 0
*c*_w_*, *γ**_w_*, r*_w_	parameters of the age-dependent mortality function for infected adults [26]	0.1, 1.5, 0.02
*s*_g_	proportional reduction in average adult lifespan due to *Wolbachia* carriage	0.1

### 2.1. Host demography and *Wolbachia* infection

The representation of host age-structure and the effects of *Wolbachia* infection on host demography is similar to that used by Hancock *et al.* [26]. A detailed description of this part of the model is provided in the electronic supplementary material and only the main elements are summarized here.

The insect life cycle is divided into larval and adult stages. Let *L*(*t,l*) be the numbers of larvae at time *t* which have been in the larval stage for time *l*. Let the probability of a larva survives until time *l* be *θ*_L_(*t,l*) which because larval mortality may be density-dependent is a function of time. Larval mortality is described by a power function, 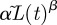, where 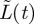 is the total larval density at time *t* and *α* and *β* are constants. The parameter α scales the quality of the habitat while higher values of the parameter *β* produce a steeper response to changing density (which we shall refer to as strong density-dependence) [19]. The duration of the larval stage is *T*_L_.

Let *A*(*t,a*) be the total number of adults of age *a* at time *t.* The probability that an adult survives until age *a* is defined as *θ*_A_(*a*) which depends on age alone. Let 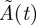 be the total number of adults at time *t* (obtained by integrating over all age classes) and *λ* the female fecundity per unit of time.

The host population is divided into classes that are either infected or uninfected by *Wolbachia,* denoted by subscripts W and U, respectively. Mating is assumed to occur at random, and the frequency of incompatible matings at time *t* is thus 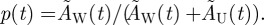 As assumed in the reaction–diffusion model, the fecundity of infected mothers is reduced by a proportion *s*_f_ while incompatible matings cause a fraction *s*_h_ of the resulting (uninfected) offspring to die. We also consider the possibility that *Wolbachia* infection may increase adult mortality, particularly in older age-classes, and the proportional reduction in average adult longevity caused by *Wolbachia* is denoted *s*_g_ [26]. Finally, we assume that *Wolbachia* may not be perfectly maternally transmitted, so that a proportion of *ω* of the offspring of infected mothers are uninfected [28].

### 2.2. Spatial population dynamics in one dimension

Host insects are distributed among *N* patches evenly spaced on a straight line. Adult insects move between the patches, and the probability of insects moving *n* patches from where they were born by the time they are of age *a* is given by the dispersal kernal *θ*_D_(*n*,*a*)*.* Using the superscript *i* to represent spatial location, the model is described by the following system of equations2.1a
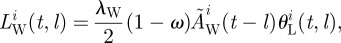
2.1b
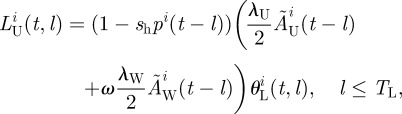
2.1c
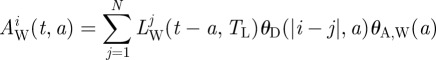
and2.1d
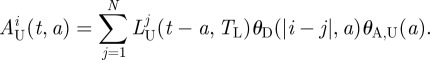


In any patch of the metapopulation, a threshold infection frequency, 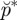, must be exceeded in order for *Wolbachia* to spread. In the special case in which the effects of host movement can be ignored (the patch experiences no immigration or emigration), Hancock *et al.* [26] showed that the unstable equilibrium infection frequency above which *Wolbachia* spreads is2.2
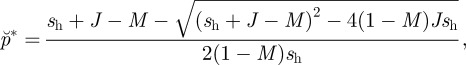


where *M* = *ω*(1 − *s*_f_) and *J* = 1 − (1 − *ω*)(1 − *s*_f_)(1 − *s*_g_). This is identical to the expression for the unstable equilibrium derived by Turelli [28] except that equation (2.2) includes a term for the effect of *Wolbachia* on adult mortality (*s*_g_). Turelli & Hoffmann's [21] reaction–diffusion model assumes that *Wolbachia* is perfectly maternally transmitted (*ω* = 0) and does not affect host lifespan (*s*_g_ = 0), which gives 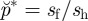. In this case, 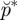 has the same interpretation as in the reaction–diffusion model, and represents the trade-off between the fitness costs of *Wolbachia* infection and the fitness advantage afforded by CI. In both models, the equilibrium condition for local spread of *Wolbachia* is perturbed by the immigration and emigration of hosts, and requires a more complex analysis [22,26,29].

### 2.3. Comparing the metapopulation model with the reaction–diffusion model

We compared the equilibrium speed of the travelling wave of *Wolbachia* infection predicted by the metapopulation equations (2.1*a*–*d*) and reaction–diffusion equation (1.1) models in the case where all patches have identical dynamics and hence equilibrium host abundance is spatially homogeneous. Host dispersal in the metapopulation is represented as a classical random walk which approaches a diffusion process as the number of patches becomes large [30].

We estimated the standard deviation of the distance between the birth place of parents and offspring (*σ* in equation (1.1)) by the average of the standard deviation of the net number of patches moved by individuals dying at age *a* weighted by the probability of death at this time (see the electronic supplementary material). Equation (1.1) assumes that *Wolbachia* does not affect host lifespan, so for the purpose of this comparison, we set *s*_g_ = 0. The average generation time of hosts in the metapopulation is *T*_L_ + *Θ*, where *Θ* is the average adult lifespan. For the parameters in table 1, *Θ* = 20 days and the average standard deviation of dispersal is *σ* = 2.7 patches per generation.

The reaction–diffusion model does not include density-dependent mortality in the host population. The effects of different forms of juvenile density-dependence on the wave speed predicted by the metapopulation model were explored by varying *β*^*i*^ across metapopulations. The second density-dependent mortality parameter, *α*^*i*^, which scales population size, was adjusted so that the equilibrium abundance of adults in the absence of *Wolbachia* was the same for each metapopulation.

### 2.4. Periodic spatial heterogeneity

To explore the effect of spatial (but not temporal) heterogeneity in larval habitat quality on the dynamics of *Wolbachia* infection, we begin by allowing the environment to vary periodically in space [31]. Larval patch quality is determined by the parameter *α*^*i*^ in the function describing density-dependent mortality with lower values of this parameter corresponding to better-quality patches that allow higher larval survival for a given density. Patch quality is assumed to switch between poor (*α*_p_) and good (*α*_g_) values with runs of good patches of length *L*_g_ being followed by runs of poor patches of length *L*_p_. This produces smooth periodic oscillations in the equilibrium adult abundance across space (see the electronic supplementary material).

We compare populations with different patterns of spatial heterogeneity in two ways. First, we keep the values of *α*_p_ and *α*_g_ the same across all metapopulations. Second, we adjust the values of these parameters so that the amplitude of the periodic spatial variation in the equilibrium abundance of adults is constant across metapopulations (details in the electronic supplementary material). In all simulations, the model is initialized by introducing *Wolbachia-*infected adults at a constant rate into the three adjacent patches at the far left of the linear array until the *Wolbachia* infection frequency in these patches reaches the upper stable equilibrium, after which the introduction ceases.

### 2.5. Spatial variability and correlation

Realistic spatial variation in the quality of larval breeding habitats will be less regular than the periodic variation described earlier. To explore this, we assume that the parameter that determines patch quality, *α*^*i*^, in the function describing density-dependent mortality is a realization of a normal random variable with mean *α*_m_ and standard deviation *σ*_m_ (table 1). We allow for the quality of nearby patches to be similar by assuming the correlation coefficient of larval habitat quality in patches separated by *n* patches is e^−*rn*^, where *r* is a measure of the spatial extent of the correlation. The spatial correlation range is defined as the maximum separation distance in number of patches for which the correlation coefficient is greater than 0.05. Spatially correlated values of *α*^*i*^ for all patches were generated by taking a Cholesky decomposition of the correlation matrix *C_ij_* = e^−*r*|*i*−*j*|^ [32]. We compare metapopulations in which the mean and standard deviation of *α*^*i*^ is the same but the range of spatial correlation varies.

### 2.6. Model parametrization

The default set of parameters used in the model runs are shown in table 1. They were chosen to represent as closely as possible a typical *Wolbachia* infection in an *A. aegypti* mosquito population (for further details, see the electronic supplementary material and Hancock *et al.* [33]). *Wolbachia* infection was assumed to cause a small reduction in average adult lifespan, with older insects experiencing enhanced mortality owing to endosymbiont carriage.

## 3. Results

### 3.1. Wave speed in a homogeneous environment

**Figure 1.**
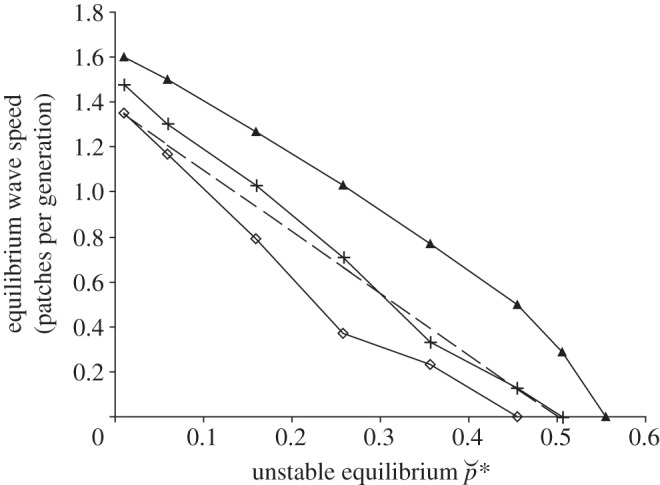
The equilibrium wave speed produced as a function of the unstable equilibrium 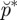. The value of 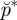 is varied by changing the reduction in adult fecundity caused by *Wolbachia* infection, *s*_f_. The form of density-dependence ranges from weak to strong as determined by the value of the parameter *β*; diamonds, crosses and triangles represent values of *β* = 0.1, 0.2 and 1.0, respectively. The dashed line is the equilibrium wave speed predicted by the reaction–diffusion model equation (1.1).

The equilibrium speed of the travelling wave of *Wolbachia* infection derived from the reaction–diffusion model equation (1.1) provides a good approximation to that of the more complex metapopulation model (figure 1). The agreement is best for low values of the unstable equilibrium infection frequency, 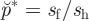 and is also affected by the form of juvenile density-dependent mortality, especially for higher 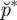. The reaction–diffusion model predicts that the speed of spread declines to zero as 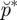 approaches 0.5; the metapopulation model indicates that spread stops at lower values of 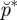 for weak density-dependence (low *β*) but the limit is above 0.5 for strong density-dependence (high *β*). Other parameters being equal; it is thus more difficult for *Wolbachia* to spread when density-dependence is weak.

The reasons why the form of juvenile density-dependence affects the spatial dynamics of *Wolbachia* are complex. The immigration of infected individuals into a patch has opposing effects on juvenile density; the introduced infected females reproduce and increase larval density, but the resident uninfected females produce fewer offspring on average due to incompatible matings with the introduced infected males. The immigration of infected individuals may thus either increase or decrease larval density depending on the rate at which they arrive and the local abundance of infected and uninfected insects. The influence of the form of density-dependence on the speed of spatial spread depends on whether immigration causes larval density to increase or decrease. If the density of larvae decreases, their mortality will be comparatively lower if density-dependence is strong. More juveniles will survive to become adults and there will be a greater number of infected adults able to disperse to other patches and initiate *Wolbachia* spread. If density-dependence is weak then the larval mortality remains relatively high even though their density is reduced. This means that fewer infected adults disperse to other patches. The effect of the form of density-dependence on spatial spread is more marked when *Wolbachia* infection incurs a higher fitness cost because a greater rate of immigration of infected insects is then required to allow spread to occur.

### 3.2. Periodic variation in larval breeding habitat

Periodic spatial heterogeneity reduces the average wave speed at which the *Wolbachia* infection spreads. To explore this consider first compare metapopulations where different length runs of good- and poor-quality patches occur periodically (with the values of the patch quality *α*_g_ and *α*_p_ being the same across metapopulations). The abundance of adults approaches spatial homogeneity when the scale of environmental heterogeneity in larval habitat is finer than the standard deviation of adult dispersal per generation, *σ*. For the parameters in table 1, the equilibrium wave speed in a homogeneous environment is approximately one patch per generation.

**Figure 2.**
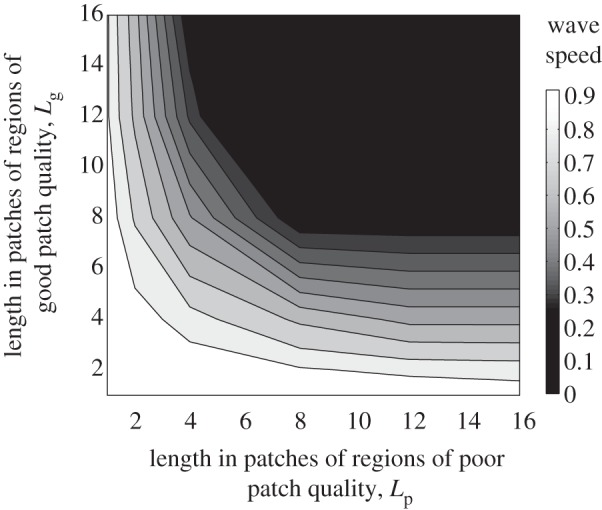
The equilibrium average speed of a travelling wave of *Wolbachia* infection (patches per generation) when larval habitat quality varies periodically. Runs of good-quality patches of length *L*_g_ are interspersed with runs of poor-quality of length *L*_p_. In good-quality patches *α*_g_ = 0.1, and in poor-quality patches *α*_p_ = 0.16.

In figure 2, wave speed is plotted as a function of the length of the runs of good- or poor-quality patches. Where adult densities do not vary spatially the wave speed remains approximately one patch per generation irrespective of the actual value of the density (as also predicted by reaction–diffusion models). This occurs when the environment is largely made up of good patches (towards the top left of figure 2) or bad patches (towards the bottom right) or where the periodicity of habitat change is short and adult movement homogenizes density (towards the bottom left).

When good- and poor-quality runs are more widely separated the wave speed markedly declines. This happens when short stretches of good patches are interspersed among longer stretches of poor patches, when the reverse occurs, and also when the length of runs of both types of patch increase together. Habitat heterogeneity thus reduces the speed with which *Wolbachia* infections can spread through a naive host population.

**Figure 3.**
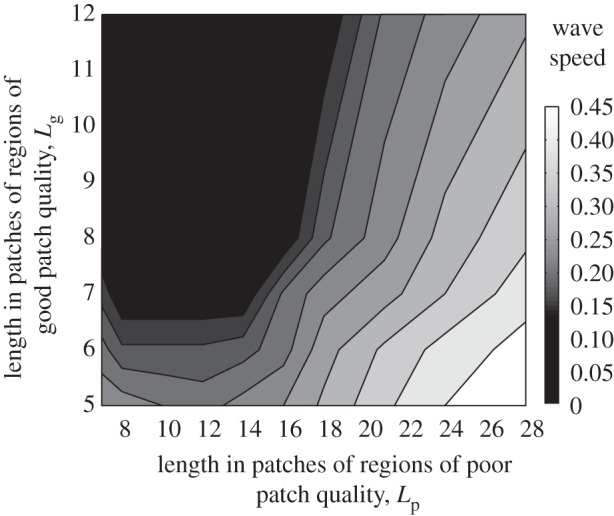
The equilibrium average speed of a travelling wave of *Wolbachia* infection (patches per generation) when larval capacity varies periodically as in figure 2 but the values of *α*_g_ and *α*_p_ are adjusted so that the amplitude of variation in adult abundance is kept constant across metapopulations (peaks are three times troughs).

Increasing the spatial scale of variation in larval patch quality increases both the amplitude and period of the variation in adult density. To separate these effects, we again alternated the quality of larval patches periodically but adjusted *α*_g_ and *α*_p_ such that the amplitude of the variation in adult density remained constant across metapopulations (figure 3). This is not possible for fine-scale variation in patch quality hence the different axes ranges in figures 2 and 3. The presence of fluctuations in adult density always substantially reduces the wave speed below that observed in homogeneous environments. Spatial spread of *Wolbachia* only occurs when the length of the runs of poor-quality patches exceeds that of the high-quality patches, and longer sequences of poor-quality patches usually produce faster wave speeds. The finest scale of heterogeneity at which the constant amplitude could be obtained involved runs of five to seven high-quality patches and seven to 12 low-quality patches (figure 3). In this region, shorter sequences of poor-quality patches lead to faster wave speeds.

**Figure 4.**
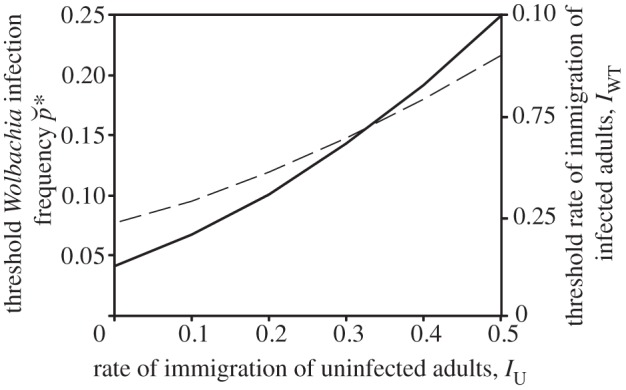
The threshold rate of immigration of infected adults required for *Wolbachia* to spread in a patch of the metapopulation (solid line), and the corresponding threshold infection frequency (dashed line), as a function of the rate of immigration of uninfected adults. Other parameters are as in table 1.

To understand why spatial variation in larval habitat quality slows the spread of infection consider the dynamics of a single patch in a metapopulation. *Wolbachia* will increase in frequency in the patch if the proportion of individuals infected exceeds the unstable equilibrium frequency 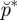 (see also §4). As the wavefront nears the patch, the rate of immigration of infected insects (*I*_W_) increases leading to this threshold being breached; however, the patch dynamics are also affected by immigration of uninfected individuals (*I*_U_). Immigration of uninfected adults (that then reproduce) increases the proportion of larvae that are uninfected and reinforces the relative recruitment of uninfected adults. The threshold rate of immigration of infected adults required for spread (*I*_WT_) and the unstable equilibrium infection frequency that is breached at this immigration rate can be calculated using a similar method to that developed by Hancock *et al.* [26] (see figure 4 and the electronic supplementary material). Both these quantities increase as the rate of immigration of uninfected adults increases (figure 4), therefore higher rates of uninfected insect immigration require a greater influx of infected individuals before *Wolbachia* can spread.

**Figure 5.**
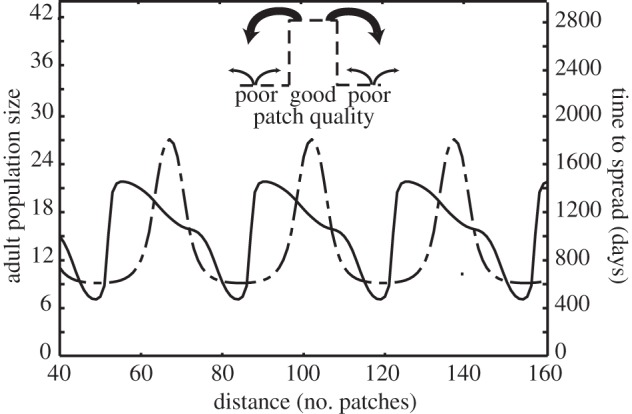
Variation in the speed of spread of *Wolbachia* infection in a periodic environment. The environment is assumed to consist of alternating long and short runs of poor and good larval patches (*L*_p_ = 28, *L*_g_ = 7) and the equilibrium distribution of adult abundance before *Wolbachia* is introduced is shown by the dot and dashed line. *Wolbachia* spreads from the left and the solid line shows the time it takes for *Wolbachia* frequency in a single patch to increase from 0.05 to the upper stable equilibrium. The inset illustrates that regions of good-quality patches supply many more immigrants to adjacent patches than regions of poor-quality patches.

The wavefront can be slowed or stopped if it approaches a region of good-quality larval patches that are a source of high rates of immigration by uninfected individuals. This is illustrated in figure 5, which shows a wave of infection moving from left to right through an environment that consists of low- and high-quality patches in the ratio 4 : 1. The local speed of spread is slowest when the wavefront spans a region of poor-quality patches but is approaching a sequence of high-quality patches. At this point, the wave produces relatively low rates of immigration of infected individuals because they are recruited in low-quality patches. The immigration of uninfected insects from patches ahead of the wave is high as they are coming from good patches. However, once the infection begins to spread in the high-quality region, the reverse effect occurs. Now the high-quality patches are producing infected immigrants and this increases the speed of the wave, especially when there are low-quality patches ahead of the wave and fewer uninfected immigrants are being produced.

Longer regions of good-quality patches provide a greater barrier for spread as they increase the rate of immigration of uninfected individuals into low-quality patches. Greater differences in quality between the two patch types also impedes spread by increasing the relative magnitude of the rate of immigration of uninfecteds into low-quality patches.

**Figure 6.**
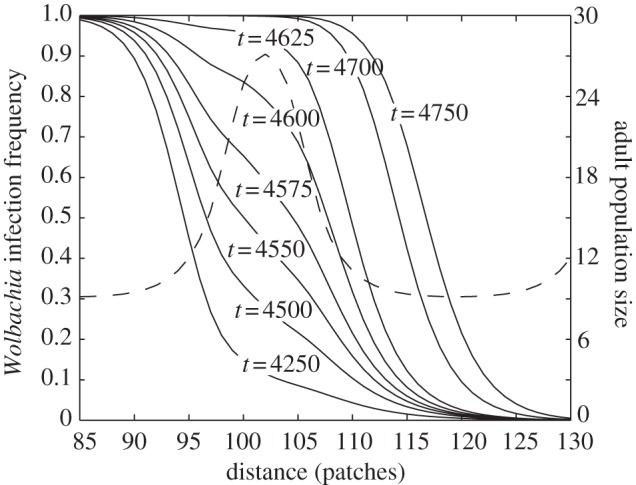
The *Wolbachia* infection frequency as a function of distance and the time in days since *Wolbachia* introduction when larval habitat quality varies periodically throughout space. The environment is assumed to consist of alternating long and short runs of poor and good larval patches (*L*_p_ = 28, *L*_g_ = 7), and the equilibrium distribution of adult abundance before *Wolbachia* is introduced is shown by the dot and dashed line. *Wolbachia* spreads from the left. Other parameters are as in table 1.

An increase in the length of the sequence of low-quality patches has two effects on the speed of spread. First, and most obviously, the average speed increases because fewer barriers of regions of high immigration of uninfected insects are encountered. But there is a second more subtle effect. In a longer region of poor patches, the wave can accelerate to its maximum speed and assume its equilibrium shape which has a steep spatial gradient in infection frequency (a steep wavefront; figure 6). This means that at the wavefront a greater fraction of the immigrants moving ahead consist of infected individuals. This assists the infection to spread into regions of high-quality patches. Therefore, the wave is less likely to be halted by barriers of high-quality patches if it encounters them after travelling through a long region of relatively poor-quality patches (figure 3). Of course, when the length of runs of low-quality patches is within the insect's dispersal range, the travelling wave can ‘jump the gap’ and hence the wave speed can increase (figure 3).

### 3.3. Spatial variability and correlation

While the study of *Wolbachia* spread through a periodic environment provides clear insight into the processes involved, real environments are more irregularly structured. To explore this, we studied movement through a metapopulation where patch qualities were chosen from a random distribution with given mean, variance and spatial covariance.

**Figure 7.**
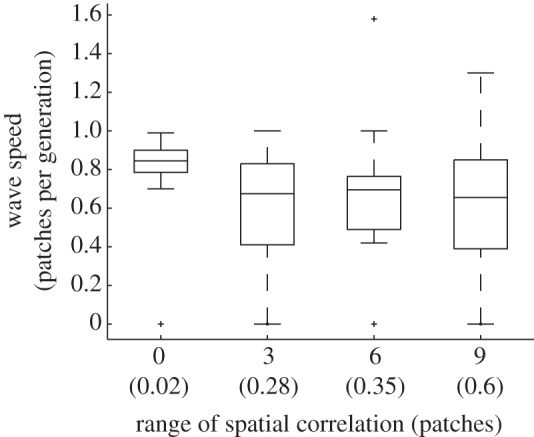
The speed of a travelling wave of *Wolbachia* infection in metapopulations where the larval habitat quality in each patch is a realization of a spatially correlated normal random process with a mean of *α*_m_ = 0.117 and standard deviation *σ*_g_ = 0.015. The box–whisker plot represents 40 realizations for each value of the correlation range. Numbers in brackets show the proportion of times that the wave halts before reaching the boundary furthest from the location of *Wolbachia* introduction. In cases where the wave halts, the wave speed over the spatial region across which *Wolbachia* attained its upper stable equilibrium frequency is plotted.

An example of a set of simulations is shown in figure 7. Keeping the mean and variance constant, increasing spatial covariance in patch quality reduced the average speed of the wave of *Wolbachia* infection and also increased the variance in speed across replicate simulations. The probability that the wave comes to a stop is also higher when there is longer-range spatial covariance in habitat quality.

Longer-range spatial covariance increases the probability that runs of low-quality patches are followed by sequences of high-quality patches which reduces the speed of the travelling wave for the reasons given in the discussion of periodic environments. In this stochastic setting, environments will occur where the travelling wave is brought to a halt by an insuperable barrier of high-quality patches, and these occur more often where habitat quality is correlated over longer distance. The variance in the speed of the wave is higher in spatially correlated environments because the wave speed increases across long regions of similar patch quality but decreases when long sequences of higher patch quality are encountered. An interesting observation is that realizations of the metapopulation occur in which the speed of spread exceeds that in a homogeneous environment when patch quality is spatially correlated (figure 7). The reason for this is that by chance these environments contain regions across which habitat quality declines, leading to long regions where adult host abundance consistently decreases in the direction the wave is travelling. Because it is easier for *Wolbachia* carried by immigrants to spread from regions of high to low density the speed of spread can be higher in these regions than when adult densities are constant. However, this effect is much weaker than the deceleration in wave speed that occurs in the opposite situation where the immigrants travel from low- to high-density regions.

## 4. Discussion

Endosymbiotic bacteria such as *Wolbachia* that spread by manipulating their hosts' reproduction can have dramatic effects on the biology of their insect hosts which can be harnessed for the management of insect pests and vectors of disease [9,34,35]. A good understanding of *Wolbachia*'s spatial epidemiology is required both to explain their distribution in natural insect communities as well as how they might be manipulated [20,21,34]. The aim of this study was to advance our understanding of these processes by developing a model that included interactions between *Wolbachia* infection frequency, host population demography and spatial variation in host habitat quality.

For appropriate limiting cases of the metapopulation model, we show that the major insights from reaction–diffusion models of *Wolbachia* spread carry over to a more complex model that incorporates realistic aspects of the host population age-structure and density-dependent regulation. In particular, there is good agreement in the conditions for spatial propagation of infection and the equilibrium speed of the travelling wave [21,23]. However, for a wide range of parameter values, details of host population ecology such as the form of density-dependent regulation can affect whether spread occurs and at what speed.

We also show that spatial heterogeneity in larval habitat quality nearly always reduces the speed at which *Wolbachia* spreads through a landscape. The reason for this is that it is difficult for an infection to spread from regions of low- to high-population density. Our analysis has shown that this is due to two main factors. First, a region of poor-quality habitat produces comparatively few migrants which makes it hard for the threshold for *Wolbachia* spread to be exceeded in an adjacent better-quality region. Second, movement of uninfected individuals from high-quality regions into lower-quality areas increases the threshold frequency that must be overcome for local *Wolbachia* establishment in these areas. This means that spatial covariance in habitat quality relative to the typical range of host dispersal is critical in determining the rate of spread of the infection. It also means that the spread of the infection can be halted within a region of low host density. Our results extend those obtained using island–mainland models of *Wolbachia* spread, which show that immigration of hosts from an uninfected mainland increases the threshold frequency required for spread on an island [22,29]. Extension of these models to describe two patches connected by asymmetric two-way migration can mimic the effects of heterogeneous host density by specifying different rates of emigration from each patch [29]. These models assume that the dynamics of *Wolbachia* depend only on their infection frequency in a host population of infinite size with discrete generations.

The same processes underlying the spatial spread of *Wolbachia* have also been associated with the spread of genetic systems involving underdominant chromosomal arrangements [23,28]. At a single location, an advantageous chromosomal form can increase in frequency, but only above a threshold frequency. Several cases where underdominant chromosomal types coexist in stable narrow ‘hybrid zones’ that coincide with regions of low population density have been documented [36–38]. These have been interpreted as regions where the spatial spread of a superior chromosomal type has become arrested because too few migrants are produced to propagate further the wave [37]. In general, dynamics of this type are expected to occur whenever spread is through a bistable wave where at a single location a threshold frequency or density must be breached before spread occurs [22,23,28,39,40].

The potential for *Wolbachia* spread to be halted by movement of uninfected insects from ahead of the wavefront has implications for the design of strategies for deliberate *Wolbachia* introductions. Releasing infected insects may not succeed in triggering *Wolbachia* spread if the liberated insects need to compete with wild-type insects for the available breeding habitat. Spread may be assisted by suppressing wild-type populations in areas that are near the release site, within the insects' dispersal range, so providing breeding habitat for the introduced insects.

In this study, our analysis of the spatial spread of *Wolbachia* was restricted to one-dimensional scenarios in order to compare the results of our metapopulation approach to the analytic results obtained from reaction–diffusion models and develop a clear understanding of the models behaviour. Barton & Turelli [22] analyse *Wolbachia* dynamics using reaction–diffusion models in one and two dimensions and show that the asymptotic speed of the travelling wave in a homogeneous environment is the same in both cases. However, fewer analytical results are available for the two-dimensional case.

Further study of *Wolbachia* spread will require a better understanding of their hosts' biology to produce more realistic models. Where the spread of *Wolbachia* or other endosymbiont infections through insect populations has been observed in nature, discrepancies between observed and predicted movement rates have been attributed, at least in part, to the spatial complexity of field populations that was not captured by the model [21,34]. In *A. aegypti* mosquitoes, the current primary targets for *Wolbachia*-based vector control interventions, larval breeding habitats can be highly heterogeneous, with a small number of sites generating a large fraction of the total recruitment to the adult population [12]. However, during rainy periods, larval breeding habitats may become more widespread [10,41].

The metapopulation model, we have developed here is a step towards incorporating the complex spatio-temporal dynamics displayed by insect host populations into models of *Wolbachia* spread. Models that aim to predict *Wolbachia* dynamics in particular host species at specific geographical locations will need to incorporate further details of the hosts' demography and will be strongly reliant on field data in order to describe the complex ecology of *A. aegypti* and other target species [42]. Our metapopulation model is purely deterministic but stochastic effects can be important in regions of low habitat quality where they are likely to have a significant influence on *Wolbachia* spread due to the small size of the local host insect population [43]. In subdivided host populations where migration is limited, local fluctuations in infection frequency may increase the likelihood of *Wolbachia* invasion [44].

There are very few insect species for which we have a good understanding of movement patterns. In this study, we used a random walk model for adult dispersal to allow comparison between the metapopulation model and the reaction–diffusion approach. For *A. aegypti* mosquitoes, Russell *et al.* [45] estimated the average lifetime dispersal to be *σ* = 78 m, in which case the range of wave speeds reported in figure 7 scales to approximately 0–46 m per generation. However, Schofield [20] showed that the form of the dispersal kernel can have a large effect on the speed of *Wolbachia* spread. Moreover, movement patterns will depend on habitat heterogeneity, and will likely be oriented towards preferred breeding habitats [46], which will influence the likelihood and speed of spread.

A priority for further study of *Wolbachia* dynamics is to combine modelling and experimental approaches to examine the demographic and behavioural processes that drive spatio-temporal fluctuations in the abundance of the insect host population [47,48], and to obtain estimates of the critical biological and environmental parameters that determine the probability and speed of invasion. An understanding of the fitness effects of *Wolbachia* on its host will be essential to this process. Our model assumes that *Wolbachia* infections have fitness costs [9,11] but there is evidence that the bacteria may increase the fitness of its host when they are infected with pathogens [49], which could help to overcome the barriers to the spatial propagation of *Wolbachia* that we have discussed [50].

## References

[1] Hilgenboecker K. Hammerstein P. Schlattmann P. Telschow A. Werren J. H. 2008 How many species are infected with *Wolbachia*?—a statistical analysis of current data. FEMS Microbiol. Lett. 281, 215–220 10.1111/j.1574-6968.2008.01110.x (doi:10.1111/j.1574-6968.2008.01110.x) 18312577 PMC2327208

[2] Laven H. 1956 Cytoplasmic inheritance in *Culex*. Nature 177, 141–142 10.1038/177141a0 (doi:10.1038/177141a0)

[3] Yen J. H. Barr A. R. 1971 New hypothesis of the cause of cytoplasmic incompatibility in *Culex pipiens*. Nature 232, 657–658 10.1038/232657a0 (doi:10.1038/232657a0) 4937405

[4] Werren J. H. 1997 Biology of *Wolbachia*. Annu. Rev. Entomol. 42, 587–609 10.1146/annurev.ento.42.1.587 (doi:10.1146/annurev.ento.42.1.587) 15012323

[5] Bian G. W. Xu Y. Lu P. Xie Y. Xi Z. Y. 2010 The endosymbiotic bacterium *Wolbachia* induces resistance to dengue virus in *Aedes aegypti*. PLoS Pathog. 6, e1000833 10.1371/journal.ppat.1000833 (doi:10.1371/journal.ppat.1000833) 20368968 PMC2848556

[6] Kambris Z. Blagborough A. M. Pinto S. B. Blagrove M. S. C. Godfray H. C. J. Sinden R. E. Sinkins S. P. 2010 *Wolbachia* stimulates immune gene expression and inhibits *Plasmodium* development in *Anopheles gambiae*. PLoS Pathog. 6, e1001143 10.1371/journal.ppat.1001143 (doi:10.1371/journal.ppat.1001143) 20949079 PMC2951381

[7] Kambris Z. Cook P. E. Phuc H. K. Sinkins S. P. 2009 Immune activation by life-shortening *Wolbachia* and reduced filarial competence in mosquitoes. Science 326, 134–136 10.1126/science.1177531 (doi:10.1126/science.1177531) 19797660 PMC2867033

[8] Moreira L. A. 2009 A *Wolbachia* symbiont in *Aedes aegypti* limits infection with dengue, chikungunya, and *Plasmodium*. Cell 139, 1268–1278 10.1016/j.cell.2009.11.042 (doi:10.1016/j.cell.2009.11.042). 20064373

[9] McMeniman C. J. Lane R. V. Cass B. N. Fong A. W. C. Manpreet S. Wang Y. O'Neill S. L. 2009 Stable introduction of a life-shortening *Wolbachia* infection into the mosquito *Aedes aegypti*. Science 323, 141–144 10.1126/science.1165326 (doi:10.1126/science.1165326). 19119237

[10] Hoffmann A. A. 2011 Successful establishment of *Wolbachia* in *Aedes* populations to suppress dengue transmission. Nature 476, 454–457 10.1038/nature10356 (doi:10.1038/nature10356). 21866160

[11] Walker T. 2011 The wMel *Wolbachia* strain blocks dengue and invades caged *Aedes aegypti* populations. Nature 476, 450–453 10.1038/nature10355 (doi:10.1038/nature10355). 21866159

[12] Jeffery J. A. L. Nguyen Thi Y. Vu Sinh N. Le Trung N. Hoffmann A. A. Kay B. H. Ryan P. A. 2009 Characterizing the *Aedes aegypti* population in a Vietnamese village in preparation for a *Wolbachia*-based mosquito control strategy to eliminate dengue. PLoS Negl. Trop. Dis. 3, e552 10.1371/journal.pntd.0000552 (doi:10.1371/journal.pntd.0000552) 19956588 PMC2780318

[13] Dye C. 1984 Models for the population dynamics of the yellow fever mosquito *Aedes aegypti*. J. Anim. Ecol. 53, 247–268 10.2307/4355 (doi:10.2307/4355)

[14] Harrington L. C. Ponlawat A. Edman J. D. Scott T. W. Vermeylen F. 2008 Influence of container size, location, and time of day on oviposition patterns of the dengue vector, *Aedes aegypti*, in Thailand. Vector Borne Zoonotic Dis. 8, 415–423 10.1089/vbz.2007.0203 (doi:10.1089/vbz.2007.0203) 18279006 PMC2978047

[15] Honorio N. A. Codeco C. T. Alvis F. C. Magalhaes M. A. F. M. Lourenco-de-Oliveira R. 2009 Temporal distribution of *Aedes aegypti* in different districts of Rio de Janeiro, Brazil, measured by two types of traps. J. Med. Entomol. 46, 1001–1014 10.1603/033.046.0505 (doi:10.1603/033.046.0505) 19769029

[16] Koenraadt C. J. M. Aldstadt J. Kijchalao U. Sithiprasasna R. Getis A. Jones J. W. Scott T. W. 2008 Spatial and temporal patterns in pupal and adult production of the dengue vector *Aedes aegypti* in Kamphaeng Phet, Thailand. Am. J. Trop. Med. Hyg. 79, 230–238 18689629

[17] Scott T. W. Morrison A. C. Lorenz L. H. Clark G. G. Strickman D. Kittayapong P. Zhou H. Edman J. D. 2000 Longitudinal studies of *Aedes aegypti* (Diptera: Culicidae) in Thailand and Puerto Rico: Population dynamics. J. Med. Entomol. 37, 77–88 10.1603/0022-2585-37.1.77 (doi:10.1603/0022-2585-37.1.77) 15218910

[18] Sheppard P. M. MacDonald W. W. Tonn R. J. Grab B. 1969 Dynamics of an adult population of *Aedes aegypti* in relation to dengue haemorrhagic fever in Bangkok. J. Anim. Ecol. 38, 661–702 10.2307/3042 (doi:10.2307/3042) PMC2554773 5307596

[19] Legros M. Lloyd A. L. Huang Y. X. Gould F. 2009 Density-dependent intraspecific competition in the larval stage of *Aedes aegypti* (Diptera: Culicidae): revisiting the current paradigm. J. Med. Entomol. 46, 409–419 10.1603/033.046.0301 (doi:10.1603/033.046.0301) 19496407 PMC2702140

[20] Schofield P. 2002 Spatially explicit models of Turelli–Hoffmann *Wolbachia* invasive wave fronts. J. Theor. Biol. 215, 121–131 10.1006/jtbi.2001.2493 (doi:10.1006/jtbi.2001.2493). 12051989

[21] Turelli M. Hoffmann A. A. 1991 Rapid spread of an inherited incompatability factor in California *Drosophila*. Nature 353, 440–442 10.1038/353440a0 (doi:10.1038/353440a0) 1896086

[22] Barton N. H. Turelli M. 2011 Spatial waves of advance with bistable dynamics: cytoplasmic and genetic analogues of allee effects. Am. Nat. 138, E48–E75 10.1086/661246 (doi:10.1086/661246) 21828986

[23] Barton N. H. 1979 The dynamics of hybrid zones. Heredity 43, 341–359 10.1038/hdy.1979.87 (doi:10.1038/hdy.1979.87)

[24] Crow J. F. Kimura M. 1970 An introduction to population genetics theory, 1st edn New York, NY: Harper & Row

[25] Nagylaki T. 1975 Conditions for the existance of clines. Genetics 80, 595–615 1232026 10.1093/genetics/80.3.595 PMC1213362

[26] Hancock P. A. Sinkins S. P. Godfray H. C. J. 2011 Population dynamic models of the spread of *Wolbachia*. Am. Nat. 177, 323–333 10.1086/658121 (doi:10.1086/658121) 21460541

[27] Rasgon J. L. Scott T. W. 2004 Impact of population age structure on *Wolbachia* transgene driver efficacy: ecologically complex factors and release of genetically modified mosquitoes. Insect Biochem. Mol. Biol. 34, 707–713 10.1016/j.ibmb.2004.03.023 (doi:10.1016/j.ibmb.2004.03.023) 15242712

[28] Turelli M. 1994 Evolution of incompatibility inducing microbes and their hosts. Evolution 48, 1500–1513 10.2307/2410244 (doi:10.2307/2410244) 28568404

[29] Flor M. Hammerstein P. Telschow A. 2007 *Wolbachia*-induced unidirectional cytoplasmic incompatibility and the stability of infection polymorphism in parapatric host populations. J. Evol. Biol. 20, 696–706 10.1111/j.1420-9101.2006.01252.x (doi:10.1111/j.1420-9101.2006.01252.x) 17305835

[30] Okubo A. Levin S. A. 2001 Diffusion and ecological problems: modern perspectives. New York, NY: Springer

[31] Shigesada N. Kawasaki K. 1997 Biological invasions: theory and practice. New York, NY: Oxford University Press

[32] Yamazaki F. Shinozuka M. 1990 Simulation of stochastic fields by statistical preconditioning. J. Eng. Mech. ASCE 116, 268–287 10.1061/(ASCE)0733-9399(1990)116:2(268) (doi:10.1061/(ASCE)0733-9399(1990)116:2(268))

[33] Hancock P. A. Sinkins S. P. Godfray H. C. J. 2011 Strategies for introducing *Wolbachia* to reduce transmission of mosquito-borne diseases. PLoS Negl. Trop. Dis. 5, e1024 10.1371/journal.pntd.0001024 (doi:10.1371/journal.pntd.0001024) 21541357 PMC3082501

[34] Himler A. G. 2011 Rapid spread of a bacterial symbiont in an invasive whitefly is driven by fitness benefits and female bias. Science 332, 254–256 10.1126/science.1199410 (doi:10.1126/science.1199410) 21474763

[35] Oliver K. M. Moran N. A. Hunter M. S. 2005 Variation in resistance to parasitism in aphids is due to symbionts not host genotype. Proc. Natl Acad. Sci. USA 102, 12 795–12 800 10.1073/pnas.0506131102 (doi:10.1073/pnas.0506131102) PMC1200300 16120675

[36] Barrowclough G. F. Groth J. G. Mertz L. A. Gutierrez R. J. 2005 Genetic structure, introgression, and a narrow hybrid zone between northern and California spotted owls (*Strix occidentalis*). Mol. Ecol. 14, 1109–1120 10.1111/j.1365-294X.2005.02465.x (doi:10.1111/j.1365-294X.2005.02465.x) 15773939

[37] Barton N. H. Hewitt G. M. 1989 Adaptation, speciation and hybrid zones. Nature 341, 497–503 10.1038/341497a0 (doi:10.1038/341497a0) 2677747

[38] Ruegg K. 2008 Genetic, morphological, and ecological characterization of a hybrid zone that spans a migratory divide. Evolution 62, 452–466 10.1111/j.1558-5646.2007.00263.x (doi:10.1111/j.1558-5646.2007.00263.x) 18039327

[39] Maini P. K. Malaguti L. Marcelli C. Matucci S. 2007 Aggregative movement and front propagation for bi-stable population models. Math. Models Meth. Appl. Sci. 17, 1351–1368 10.1142/s0218202507002303 (doi:10.1142/s0218202507002303)

[40] Wang M. H. Kot M. Neubert M. G. 2002 Integrodifference equations, Allee effects, and invasions. J. Math. Biol. 44, 150–168 10.1007/s002850100116 (doi:10.1007/s002850100116) 11942530

[41] Azil A. H. Long S. A. Ritchie S. A. Williams C. R. 2010 The development of predictive tools for pre-emptive dengue vector control: a study of *Aedes aegypti* abundance and meteorological variables in North Queensland, Australia. Trop. Med. Int. Health 15, 1190–1197 10.1111/j.1365-3156.2010.02592.x (doi:10.1111/j.1365-3156.2010.02592.x) 20636303

[42] Magori K. Legros M. Puente M. E. Focks D. A. Scott T. W. Lloyd A. L. Gould F. 2009 Skeeter buster: a stochastic, spatially explicit modeling tool for studying *Aedes aegypti* population replacement and population suppression strategies. PLoS Negl. Trop. Dis. 3, e508 10.1371/journal.pntd.0000508 (doi:10.1371/journal.pntd.0000508) 19721700 PMC2728493

[43] Jansen V. A. A. Turelli M. Godfray H. C. J. 2008 Stochastic spread of *Wolbachia*. Proc. R. Soc. B 275, 2769–2776 10.1098/rspb.2008.0914 (doi:10.1098/rspb.2008.0914) PMC2605827 18755670

[44] Reuter M. Lehmann L. Guillaume F. 2008 The spread of incompatibility-inducing parasites in sub-divided host populations. BMC Evol. Biol. 8, 134 10.1186/1471-2148-8-134 (doi:10.1186/1471-2148-8-134) 18460188 PMC2396168

[45] Russell R. C. Webb C. E. Williams C. R. Ritchie S. A. 2005 Mark–release–recapture study to measure dispersal of the mosquito *Aedes aegypti* in Cairns, Queensland, Australia. Med. Vet. Ent. 19, 451–457 10.1111/j.1365-2915.2005.00589.x (doi:10.1111/j.1365-2915.2005.00589.x) 16336310

[46] Wong J. Stoddard S. T. Astete H. Morrison A. C. Scott T. W. 2011 Oviposition site selection by the dengue vector *Aedes aegypti* and its implications for dengue control. PLoS Negl. Trop. Dis. 5, e1015 21532736 10.1371/journal.pntd.0001015 PMC3075222

[47] Ellis A. M. 2008 Linking movement and oviposition behaviour to spatial population distribution in the tree hole mosquito *Ochlerotatus triseriatus*. J. Anim. Ecol. 77, 156–166 10.1111/j.1365-2656.2007.01319.x (doi:10.1111/j.1365-2656.2007.01319.x) 18177335

[48] Vinatier F. Tixier P. Duyck P. Lescourret F. 2011 Factors and mechanisms explaining spatial heterogeneity: a review of methods for insect populations. Methods Ecol. Evol. 2, 11–22 10.1111/j.2041-210X.2010.00059.x (doi:10.1111/j.2041-210X.2010.00059.x)

[49] Hedges L. M. Brownlie J. C. O'Neill S. L. Johnson K. N. 2008 *Wolbachia* and virus protection in insects. Science 322, 702 10.1126/science.1162418 (doi:10.1126/science.1162418) 18974344

[50] Fenton A. Johnson K. N. Brownlie J. C. Hurst G. D. D. 2011 Solving the *Wolbachia* paradox: modeling the tripartite interaction between host, *Wolbachia*, and a natural enemy. Am. Nat. 178, 333–342 10.1086/661247 (doi:10.1086/661247). 21828990

